# The role of arthroscopy in the management of adult elbow trauma

**DOI:** 10.1186/s40634-023-00710-z

**Published:** 2023-12-22

**Authors:** Maria Valencia, AM. Foruria

**Affiliations:** 1 Division of Shoulder and Elbow Surgery, Hospital Universitario Fundación Jiménez Díaz, Avenida Reyes Católicos Nº2, Madrid, 28040 Spain; 2 Head of Division of Shoulder and Elbow Surgery Unit, Hospital Universitario Fundación Jiménez Díaz, Avenida Reyes Católicos Nº2, Madrid, 28040 Spain

**Keywords:** Elbow Arthroscopy, Elbow Trauma, Coronoid fracture, LCL repair

## Abstract

Fractures around the elbow are often challenging to treat and in most cases require an extensive approach. Since the development of elbow arthroscopy, most authors have pointed out the potential advantages of a less invasive technique that can be useful for visualization and reduction of the articular fragments with an eventual percutaneous fixation. Arthroscopic techniques provide a limited exposure that may lead to a faster wound healing, lower rate of complications and thus, better recovery of range of motion. However, elbow arthroscopy is also a demanding technique, especially in a swollen and fractured joint, and it is not exempt of risks. The overall rate of complications has been rated from 1.5% to 11% and nerve injury rates from 1.26–7.5%.

The objective of this review is to present the arthroscopic setup and general surgical technique for the management of elbow trauma and to define some clear indications. Patient positioning and operating room display is key in order to obtain success. In addition to the arthroscopic equipment, fluoroscopy is almost always necessary for percutaneous fixation and precise preparation is mandatory. In the last decade, literature regarding new portals or surgical tips for arthroscopic treatment of elbow fractures have been published.

The main indications for fracture arthroscopic-assisted fixation are those articular fractures involving the coronoid, distal humerus shear fractures in the coronal plane (trochlear and capitellum fractures) and, more controversially, those affecting the radial head. The treatment of these type of fractures all arthroscopically is exponentially demanding as it might also require ligament repair. For coronoid fractures, it can be useful in Morrey type II and III, and O´Driscoll anteromedial facet fractures associated to a posteromedial instability pattern that also require a repair of the LCL. Although excellent results have been published, comparative series are scarce. Radial head fractures can also be approached arthroscopically in simple non-comminute fractures that can be fixed percutaneously.

In conclusion, arthroscopy of the elbow is an excellent tool to better understand and visualize articular fractures of the elbow. However, despite the advances in surgical technique, whether it improves clinical and radiological results is still to be proven.

## Introduction

Fractures around the elbow joint are often challenging to treat as they usually require extended approaches and may put at risk neurovascular and ligamentous structures. In the majority of the cases it is mandatory to carry out a conventional open approach in order to assess fractures that affect several bones or require hardware implantation and/or ligament repair or reconstruction. On the other hand, there are other cases that might benefit from arthroscopy to help in achieving an anatomical reduction of articular surface and to perform a percutaneous fixation after reduction. It also allows for an evaluation of concomitant intra-articular injuries, such as loose bodies and chondral lesions, to limit the risk of heterotopic ossification compared to open surgery.

The less extensive approach provided by the arthroscopic technique has some inherent advantages such as limited soft tissue damage than might allow for an earlier mobilization and potentially less stiffness in the mid-term follow-up. However, elbow arthroscopy is a demanding technique that requires a thorough knowledge of the normal anatomy and nerve distribution. Moreover, advanced skills are also needed in order to perform a safe percutaneous fixation avoiding nerve and vascular damage.

From the current literature, we can define clear indications for elbow arthroscopy, for example, the coronoid fractures. It is especially recommended for those affecting the anteromedial facet that would require a repair of the LCL and might need otherwise a double open approach [1–4]. Several authors have also reported good results in lateral condyle fractures or coronal shear fractures of the distal humerus [29]. The results of the radial head fixation have also been published in small case series with satisfactory results [13–25]. More controversial is the arthroscopic repair of the LCL [27].

## Arthroscopic setup

Elbow arthroscopy setup requires specific experience of the surgical team. It is usually performed in the lateral decubitus with the elbow placed on a padded holder at 90º of shoulder forward flexion and internal rotation, but the patient can also be positioned in the prone or supine position. A regional block can be performed before or after surgery if the surgeon wants to perform an immediate postoperative neurovascular examination, and it can be combined with a general anaesthesia [16].

**Fig. 1:**
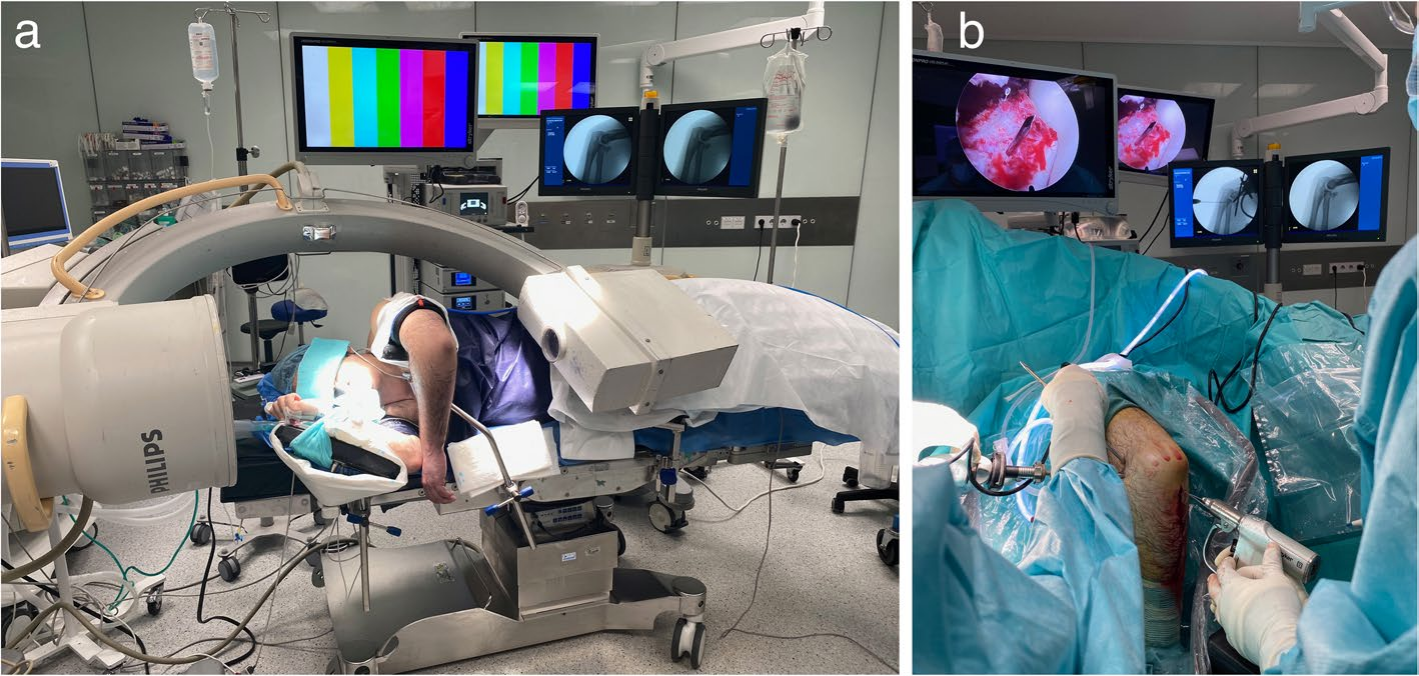
**A** Fluoroscopy is brought to the field from the head of the patient, with the arc tilted 40–60 degrees above the patient for screen visualization, and the thicker x-ray beam detector near the patient´s head. This disposition allows to work in the reduction and fixation at the same time the images are obtained. **B** The field is then prepared in a sterile fashion over the patient and the radioscopy device

It is important to confirm preoperatively that fluoroscopy can be located in the OR without interference of the arthroscopic equipment as it is determinant for percutaneous fixation. (Fig. 1). Fluoroscopy is positioned before draping ensuring enough space is available to perform the surgery while arthroscopy and fluoroscopic screens are visible. A tourniquet inflated to 250mmHG is routinely used.

Location of the portals should be carefully planned as the anatomical landmarks might be difficult to identify due to the swelling and the distorted bony references. In the acute setting, entrance to the joint is usually straightforward as it is distended by hematoma, so the injection of saline is not mandatory. A standard 4 mm 30º scope is routinely used, but a 70º camera could be useful for coronoid and capitellar fractures. The use of pump is not recommended and it is preferable to let the fluid come into the joint by gravity after hanging the saline bags 2 m above the ground level. No suction is applied to the instruments to avoid articular space to collapse or soft tissue damage with the motorized devices.

Apart from the camera, other instruments should be prepared for the surgery; a synoviotome and a radiofrequency electrode system are used for cleaning the joint and the fracture site. Wissinger rods are useful for capsular retraction and fracture manipulation. Small periosteal elevators or other flat and long instruments are very useful assisting in reduction and holding fracture fragments in place. A grasper is also used for fragment management and holding provisional wires in place.

## Surgical technique

Surgical steps are summarized in Table 1. Independently of what fracture needs to be fixed, the surgical technique is similar in every case, with variations in the portals used for the camera, the retractors and the fixation devices. After thoroughly cleaning the joint from hematoma and fracture debris, debridement of the fracture site is performed in order to allow anatomical reduction and a correct visualization of the wires used for percutaneous fixation.

**Table 1:** Surgical steps in arthroscopic fixation of elbow fractures

Surgical steps in arthroscopic fixation of elbow Fractures
1. Landmarks drawing for portal positioning
2. Camera, retractor and synoviotome insertion inside the joint
3. Articular cleaning (hematoma, fracture debris)
4. Fracture site debridement
5. Fracture reduction and provisional fixation with wires
6. Cannulated drilling
7. Definitive screw fixation

**Fig. 2:**
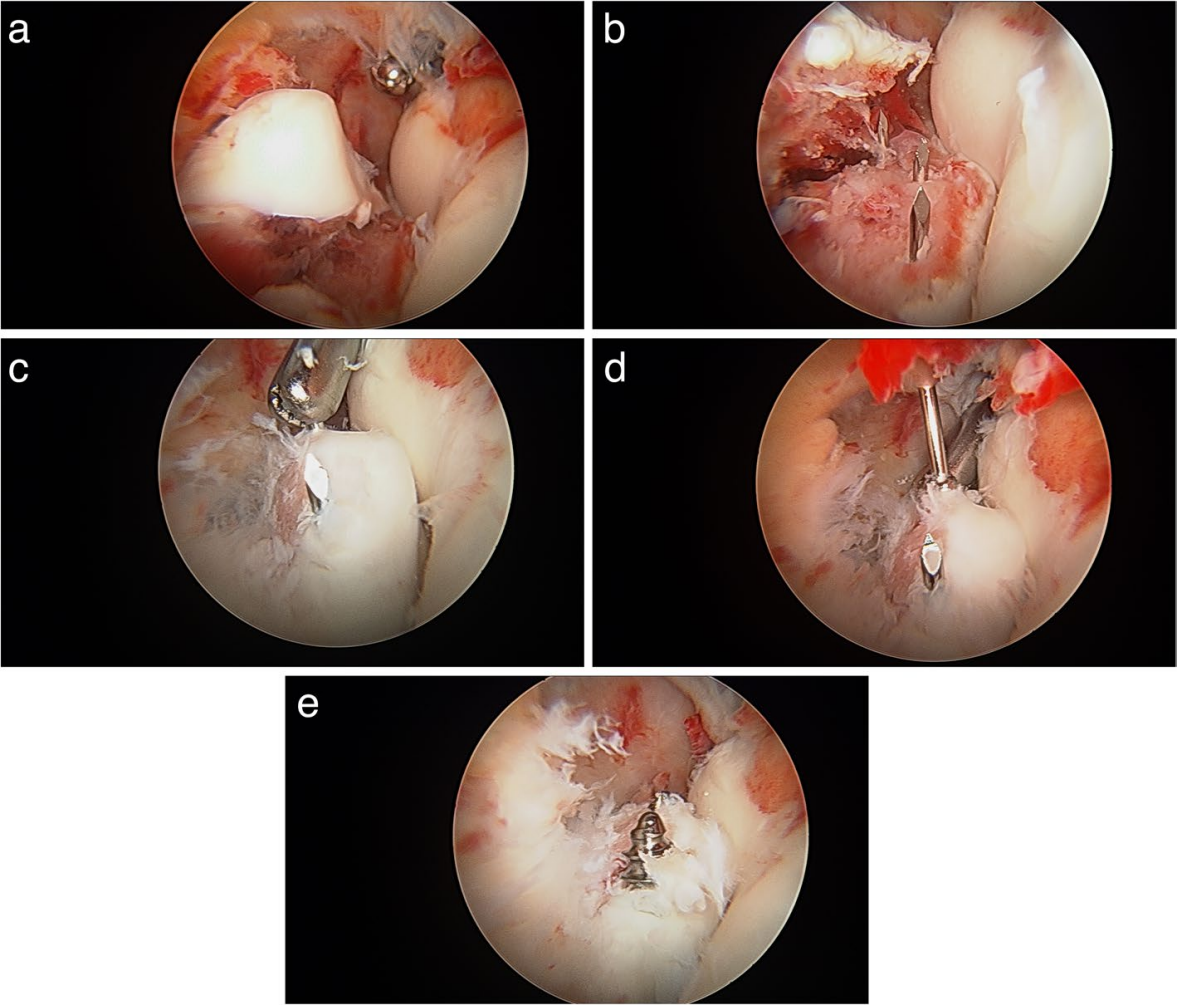
Arthroscopic coronoid fixation **A** Fracture bed is cleaned. **B** Provisional wires are introduced trough the fracture bed, perpendicular to the fracture plane. **C** The fragment is reduced and the wires are passed through it. **D** Cannulated drilling. **E** Screw placement

**Fig. 3:**
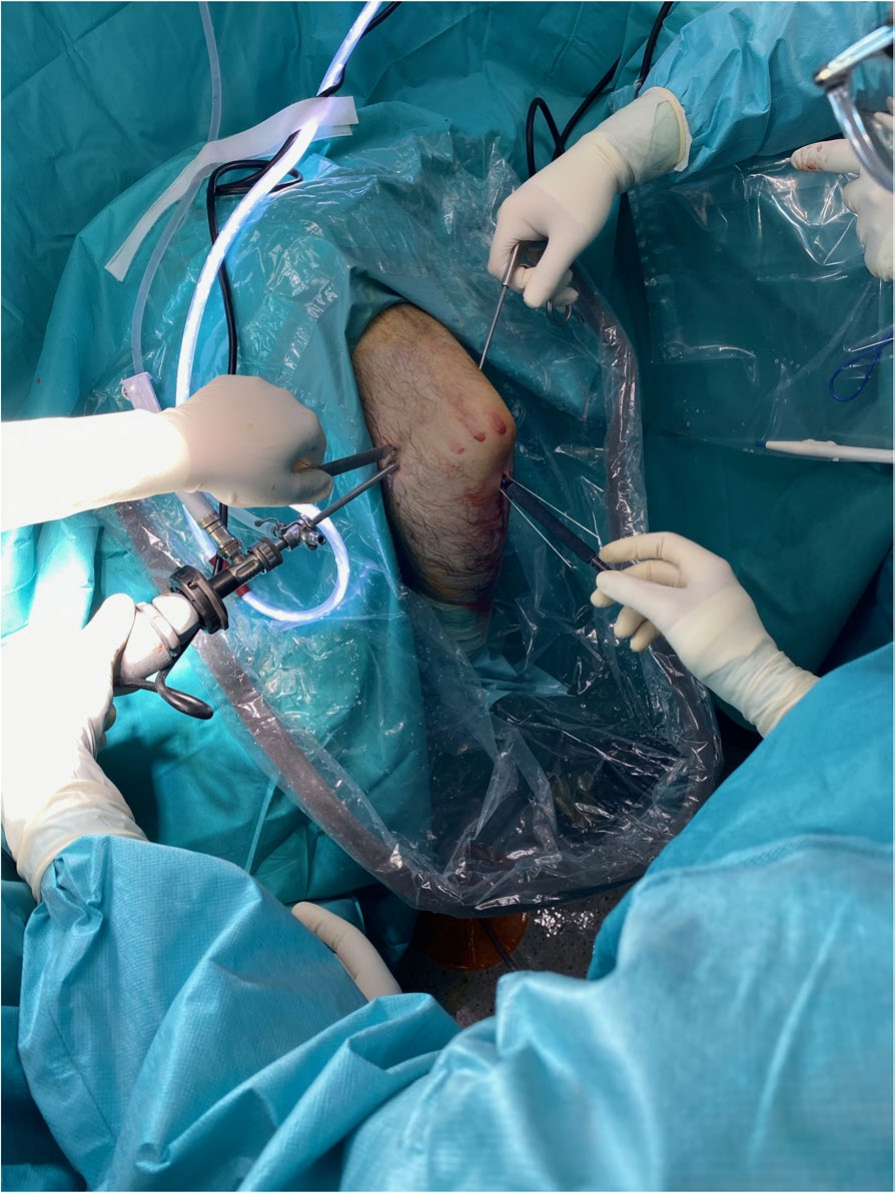
This image shows an exterior view at the moment of screw coronoid fixation. The surgeon takes the camera with the left hand and proceeds with screw length measurement with the right hand. An assistant, in the left side of the surgeon, maintains the capsule pushed anteriorly with an elevator introduced in the proximal anterolateral portal. Another assistant, in the right side of the surgeon, holds the reduction with a grasper taking a protruding reduction wire

First, fine wires are introduced trough the bone perpendicular to the fracture plane, either through the arthroscopic portals or incisions created for this purpose; these wires should aim the fracture bed and exit in the desired locations for fixation. Then, the wires are pulled back flush to the fracture bed to allow fracture reduction with the aid of blunt instruments and graspers, with both visual and radioscopic confirmation. Pressing the fracture fragment towards the fracture bed is usually enough to allow provisional k-wires to be further introduced to maintain the reduction. When performing screw fixation, based on the fragment size, we recommend introducing multiple fine k wires to maintain the reduction of the fragment and to allow cannulated drilling. The extra provisional K-wire avoids losing the reduction, especially in the common situation in which the screw guiding wire is unintentionally removed when removing the cannulated drill, or during screw insertion of solid screws. A grasper on the tip of the wire at its exit point from the bone is useful to avoid both wire or fragment migration while drilling and screwing phases. The length of the screw is then measured with the aid of another wire of the same length, subtracting the length of the proximal part of the wire outside the bone. Finally, a cannulated or solid screw is introduced, reproducing this technique with as many screws as needed (Figs. 2 and 3).

## Indications and results

### Coronoid fractures

The most commonly reported treatment of elbow fractures assisted by arthroscopy is the coronoid fracture [1–4].

Indications for fixation of coronoid fractures are equivalent as for the open procedure [9]: Fractures: Morrey type II and III, and O´Driscoll anteromedial facet fractures associated to a posteromedial instability pattern that also require a repair of the LCL.

For isolated bony coronoid fractures (in absence of other fractures) standard anteromedial viewing portal is performed, as well as the anterolateral and proximal anterolateral portals for the use of retractors and reduction tools. In the case of coronoid fixation, introducing the camera from one of the lateral portals allows a wider visualization of the coronoid and the lateral fracture bed. On the other hand, when there is a distortion of normal anatomy, a posterior trans articular portal has been suggested by Kim et al. [17]. The percutaneous fixation is performed from the subcutaneous border of the proximal ulna in a posterior to anterior direction [21]. Several techniques have been described: screws, suture lasso technique or indirect capsular sutures [11]. Some authors recommend the use of an anterior cruciate ligament guide in order to position the k wires but it can also be performed freehand under direct arthroscopic visualization [1]. The level of the insertion can be inferred using the radial head as a reference. There is controversy on whether to use cannulated 2.5 mm screws but solid 2.7 mm screws might provide a stronger fixation and are the preferred author´s technique [10]. The thinner screws are more difficult to find in the market in the necessary length to provide coronoid fixation without the need of sinking the whole screw pass the proximal cortical. Solid screws are inserted after drilling with a cannulated drill over a guiding wire, which is removed for screw insertion. Measurement should be carefully performed so that the length is enough to fix the fragment but the tip does not penetrate excessively in the joint interfering with elbow flexion. An indirect fixation by means of sutures can also be performed. A curette or a blunt instrument might be used to help maintaining reduction.

Adams et al. published good results in their 7 cases series of isolated coronoid fracture arthroscopic fixation [1]. Colozza et al. [4] also reported excellent results of anteromedial fractures. Of 36 cases, 27 required a repair of the LCL and 8 of them were performed in an arthroscopic fashion. However, literature comparing both open and arthroscopic techniques is scarce. Oh et al. [23] published the results of a case control study including 25 fractures (10 treated by open surgery and 15 arthroscopically). They did not find any differences in terms of radiological or clinical results but they describe a higher rate of complications in the open group (40% vs 13%) with two cases of ulnar nerve neuropathy.

### Radial head fracture

The fixation of the radial head by means of arthroscopy is yet controversial as it has not demonstrated greater clinical or radiological results than the open technique [14]. Indications for arthroscopy would include resection of less than 30% fragments [20] and fixation of simple non comminute fractures of the radial head [20–25].

In these cases, a posterolateral viewing portal can be established first as visualization portal and the soft spot portal as a working portal. If the fragment is anteriorly located as it is usually the case, an anteromedial portal could be useful for viewing and fracture reduction and fixation can be performed from the lateral side [8] playing with radius rotations for proper screw orientation.

Michels et al. described a technique in prone position with the arm flexed 90º [20]. A mid lateral portal is used for visualization and the anterolateral portal for instrumentation. Pronation and supination can help in identifying and defining the fragments of the fracture. A probe or a k wire can be percutaneously inserted and used in a joystick fashion to help in reduction manoeuvres and for reduction itself. A small stab incision is recommended to introduce the screws. On the other hand, a standard technique in lateral decubitus and starting from the anteromedial portal can be performed. The soft spot is then used to introduce a periosteal elevator in order to help in reduction and fixation is performed through the anterolateral portal [6, 7]. Care should be taken when performing a percutaneous fixation, as posterior interosseous nerve is close in this location. The fixation can be performed with cannulated screws in a similar fashion to the open technique and the size of the screws can be checked under fluoroscopy.

The results of this technique have been published in two case series by Rolla et al. [25] and Michels et al. [20]. Michels et al. obtained excellent results for a series of 16 Mason Type II fractures without reporting any complications.

### Capitellar fractures

Isolated capitellar fractures are infrequent and represent 1% of all elbow fractures and in more than 60% of the cases are associated to fractures of the radial head or LCL injuries. Several open approaches have been described from the lateral Kocher and Kaplan to some anterior limited approaches with satisfactory results [3].

When performing an arthroscopic capitellar fixation, fractures can be reduced in a closed fashion before starting arthroscopy or once the scope is located inside the joint. The fractures typically reduce with full extension of the elbow and the forearm supinated. Then, the elbow should be flexed gently and maintaining the traction so that the radial head does not displace the fragment again [22]. Once the fragment is reduced, the elbow should be flexed more than 90º to keep the fragment in place. Conventional portals can be used but in cases of plastic deformity of the distal humerus, or comminute fractures, posterolateral portals can be useful to debride. Bryan- Morrey type I fractures (large osseous piece of the capitellum involved) can be fixed with 3.5 mm screws from posterior to anterior through a small incision in the lateral triceps and a minimum of two screws are recommended depending on the fragment size [30]. Bryan-Morrey types II and III fractures can be managed by osteosutures and very small fragments can be excised [8]. Type IV fractures (McKee modification that includes trochlear extension) are usually fixed with three screws in a tripod fashion, with one screw in the medial trochlea and two in the capitellum.

Clinical and radiological results have been reported in some cases series [15–29]. The most recent, by Zhang et al. included 10 cases, and reported good clinical and radiological results and no complications related to the surgical technique. There were 4 cases of AVN with no clinical impact. The authors recommend to wait in between 3 to 14 days to undergo the surgery to avoid excessive bleeding during the scope but they did not relate any association between time from injury to surgery and incidence of AVN.

However, comparative series are still lacking in order to determine if the advantages of the arthroscopic technique are substantial.

### Ligament repair

In order to treat complex coronoid and radial head fractures, traditionally, an open approach was advocated for the treatment of the ligamentous lesions that are present in these injuries [26]. However, more recently, an arthroscopic management of injuries to the LUCL has been reported [5–27]. In the acute setting, when the ligament is torn, a formal repair with anchors can be performed, usually in the proximal attachment [5].

The formal repair can be performed arthroscopically viewing primarily the joint from the posterior compartment.

As the first step, the lateral epicondyle should be debrided and decorticated in order to expose subchondral bone and enhance healing. Then, the isometric point should be identified in order to place the anchor with the help of a needle entering straight lateral to the bone. Depending on the type of anchor used, a drill guide might be necessary as well. The third step should be to pass the sutures through the proximal part of the ligament with an indirect suture shuttling device. However, the results of this technique have not been published yet to our knowledge.

## Authors opinion and recommendations

We recommend arthroscopic fixation for elbow fractures in cases in which this technique is going to save an open approach. In other words, it does not make sense to fix the radial head arthroscopically if an open lateral approach is also going to be performed for lateral collateral ligament repair. For coronoid fractures, we do not perform arthroscopic fixation of the injuries in terrible triads, as the coronoid fragment is usually small and does not need to be fixed in the majority of cases. Standard radial head repair/replacement along with lateral collateral ligament repair is often enough, and in the rare cases of persistent instability, open medial collateral ligament repair with or without coronoid fixation or reconstruction is performed. Coronoid fractures in the setting of trans-ulnar fracture dislocations are also fixed open, as the proximal ulna needs to be exposed for fixation. However, arthroscopy is most useful in cases of isolated coronoid fractures needing fixation (those greater than 50% of the height of the coronoid or associated to persistent ligamentous instability), along with lateral collateral ligament repair. The contraindications for this technique include: 1) sublime tubercle involvement, which needs in our opinion buttress plate fixation; 2) comminution, there must be a big competent fragment amenable for fixation, as opposed to multiple small fragments; and 3) the fragment should be wide and big enough as to be fixed at least by two screws.

For distal humerus coronal shear fractures, arthroscopic fixation is possible when the following is fulfilled: 1) The fracture should be able to be reduced closed, or at least with the aid of scope visualization and retractors or rods; 2) there should be only one fragment, or at least the majority of the fracture should be included in a big fixable fragment. Fixation will be performed from posterior to anterior, holding the reduction with a grasper and with the aid of elbow flexion entrapping the fragment between the fracture bed and the radial head. Isolated, one fragment, capitellar fractures are ideal for arthroscopic fixation. Capitellar fractures with trochlear extension in a single fragment are also good candidates. Osteochondral fragments can be debrided or arthroscopically fixed with Crossed PDS sutures depending on size and location, being amenable for fixation big fragments in the central part of the capitellum. Severe capitellar comminution can be debrided or treated conservatively depending on the existence of mechanical interference with complete range of motion after intra-articular anaesthesia injection.

Radial head fractures are the most controversial indication in our hands. Non displaced Mason type I fractures are treated conservatively. Mason type III and IV are too complex for arthroscopic fixation in our hands, either because of the presence of severe displacement, a instable neck fracture, comminution, associated injuries needing open repair (i.e. ligament ruptures or translunar fractures), or a combination of the above. Type II radial head fractures would be suitable for arthroscopic fixation, but the majority can be treated conservatively (those without mechanical interference in exam under intra-articular anaesthesia), many are anterior in location (needing fixation from posterior, increasing the chances of reduction loss and decreasing the strength of fixation due to the posterior location of the compression screw mechanism; or fixation in full pronation or supination for fixation through lateral or medial portals to avoid anterior percutaneous approaches, decreasing the control of the reduction); and, as mentioned before, arthroscopic radial head fixation does not seem to further improve the good results of open repair. Having said that, skilled elbow surgeons will find this technique very rewarding for specific easy cases.

## Conclusions

Arthroscopy of the elbow has evolved significantly in the last decade, becoming an excellent tool to better understand and visualize some articular fractures of the elbow. Its main advantage is preserving the soft tissue envelope and avoidance of multiple approaches in specific complex elbow instability patterns. However, despite the advances in surgical technique, whether it improves clinical and radiological results is still to be proven.

## Abbreviations

LCL: Lateral Collateral Ligament
OR: Operating Room
